# 2-Hy­droxy­anilinium 3,5-dinitro­benzoate

**DOI:** 10.1107/S1600536812014973

**Published:** 2012-04-25

**Authors:** Qun Zhao

**Affiliations:** a National First-Class Key Discipline for Traditional Chinese Medicine of Nanjing University of Chinese Medicine, Nanjing 210046, People’s Republic of China

## Abstract

In the title molecular salt, C_6_H_8_NO^+^·C_7_H_3_N_2_O_6_^−^, which crystallizes in the chiral monoclinic space group *P*2_1_, the achiral components assemble by three different N—H⋯O, one O—H⋯O and one C—H⋯O hydrogen bonds into two-stranded chains running parallel to [010]. The dihedral angles between the carboxy group and the two nitro groups and the mean plane of their attached benzene ring are 24.5 (9), 6.1 (6) and 13.0 (1)°, respectively..

## Related literature

For background to supra­molecular structures and hydrogen bonding, see: Burrows (2004 ▶); Desiraju (2002 ▶); Steiner (2002 ▶). For related structures, see: Wang *et al.* (2008 ▶). 
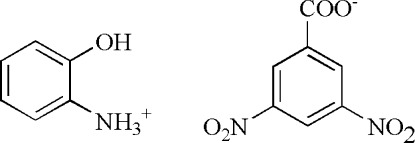


## Experimental

### 

#### Crystal data

C_6_H_8_NO^+^·C_7_H_3_N_2_O_6_^−^
*M* *_r_* = 321.25
Monoclinic, 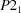

*a* = 9.4988 (19) Å
*b* = 6.0803 (12) Å
*c* = 12.109 (2) Å
β = 95.21 (3)°
*V* = 696.5 (2) Å^3^
*Z* = 2
Mo *K*α radiation
μ = 0.13 mm^−1^
*T* = 297 K
0.35 × 0.22 × 0.20 mm

#### Data collection

Rigaku Mercury2 diffractometer
Absorption correction: multi-scan (CystalClear; Rigaku, 2005 ▶) *T*_min_ = 0.967, *T*_max_ = 0.975
7134 measured reflections
1733 independent reflections
1209 reflections with *I* > 2σ(*I*)
*R*_int_ = 0.098

#### Refinement

*R*[*F* ^2^ > 2σ(*F* ^2^)] = 0.084
*wR*(*F* ^2^) = 0.218
*S* = 1.05
1733 reflections
210 parameters
1 restraint
H-atom parameters constrained
Δρ_max_ = 0.38 e Å^−3^
Δρ_min_ = −0.28 e Å^−3^

### 

Data collection: *CrystalClear* (Rigaku, 2005 ▶); cell refinement: *CrystalClear*; data reduction: *CrystalClear*; program(s) used to solve structure: *SHELXS97* (Sheldrick, 2008 ▶); program(s) used to refine structure: *SHELXL97* (Sheldrick, 2008 ▶); molecular graphics: *SHELXTL* (Sheldrick, 2008 ▶); software used to prepare material for publication: *SHELXTL*.

## Supplementary Material

Crystal structure: contains datablock(s) I, global. DOI: 10.1107/S1600536812014973/qk2032sup1.cif

Structure factors: contains datablock(s) I. DOI: 10.1107/S1600536812014973/qk2032Isup2.hkl

Supplementary material file. DOI: 10.1107/S1600536812014973/qk2032Isup4.cdx

Supplementary material file. DOI: 10.1107/S1600536812014973/qk2032Isup4.cml

Additional supplementary materials:  crystallographic information; 3D view; checkCIF report

## Figures and Tables

**Table 1:** Hydrogen-bond geometry (Å, °)

*D*—H⋯*A*	*D*—H	H⋯*A*	*D*⋯*A*	*D*—H⋯*A*
O7—H8⋯O1^i^	0.82	1.78	2.603 (7)	179
N3—H9⋯O1^ii^	0.89	1.90	2.771 (8)	168
N3—H10⋯O7^iii^	0.89	2.02	2.870 (8)	159
N3—H11⋯O2^i^	0.89	1.89	2.763 (8)	166
C12—H7⋯O1^ii^	0.93	2.53	3.240 (9)	134

Symmetry codes: (i) 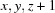; (ii) 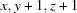; (iii) 
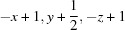
.

**Fig. 1.**
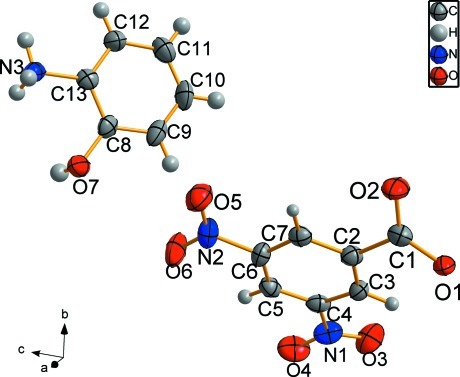
View of asymmetric unit with atom labels and displacement ellipsoids for non-H atoms drawn at the 50% probability level.

**Fig. 2.**
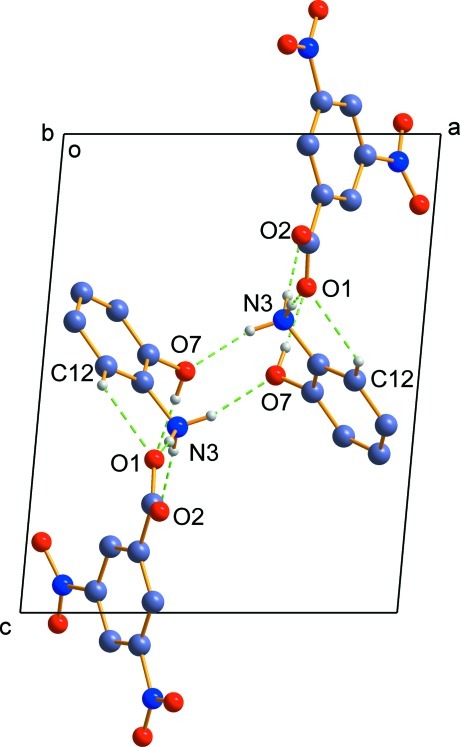
A two-stranded hydrogen bonded chain viewed along the chain direction [010]. Hydrogens not involved in hydrogen bonds are omitted for clarity. Hydrogen bonds are shown by dashed lines.

## supplementary crystallographic information

### Comment

The synthesis of multicomponent organic substances is of interest for obtaining supramolecular materials with potentially useful applications. In synthesizing such materials it is important to consider that the components contain complementary functional groups to build up links by suitable supramolecular interactions (Desiraju, 2002; Burrows, 2004). A very general way to achieve this goal is to employ components containing several matching functional groups with good hydrogen bond donor and acceptor capabilities combined with suitable backbones that bear the functional groups (Steiner, 2002). Following this strategy the title compound was synthesized from 2-hydroxyaniline and 3,5-dinitrobenzoic acid and was studied by X-ray diffraction. It is a proton-transfer salt, C_6_H_8_NO^+^.C_7_H_3_N_2_O_6_^-^, that is built up from two achiral components but crystallizes in the chiral space group *P*2_1_ with one molecule of a 2-hydoxyanilinium cation and one molecule of a 3,5-dinitrobenzoate anion in the asymmetric unit (Fig. 1). The protonized amino group (—NH_3_^+^), which is a good hydrogen-bond donor (Wang *et al.* 2008), donates two hydrogen bonds to the carboxylate oxygen atoms O1 and O2 of two adjacent 3,5-dinitrobenzoate anions and to the hydroxy oxygen O7 of another 2-hydoxyanilinium cation (Table 1). The hydroxy group of the 2-hydoxyanilinium cation in turn donates a hydrogen bond to a carboxylate oxygen O1 of a third 3,5-dinitrobenzoate anion. In this way a two-stranded infinite hydrogen bond chain is formed along [010], as shown in Fig. 2. This chain is reinforced by a weak intra-chain C—H···O bond (last entry in Table 1). The chain has Z-shaped cross section (Fig. 2). The mutual coherence between these chains is provided by van der Waals interactions, which involve also nitro oxygen atoms. Ring-ring π-π stacking contacts are missing in this structure.

### Experimental

The title compound was obtained by room temperature evaporation of a methanol solution containing 2-aminophenol and 3,5-dinitrobenzoic acid in stoichiometric 1:1 amounts.

### Refinement

All H atoms were placed in calculated positions with C—H = 0.93 Å, O—H = 0.82 Å, and N—H = 0.89 Å, and were refined as riding with *U*_iso_(H) = 1.2*U*_eq_(C) and *U*_iso_(H) = 1.5*U*_eq_(N or O). and refined in riding mode. Owing to insignifant anomalous dispersion effects the absolute structure could not be determined and the 1429 Friedel pairs were merged in the final refinement with all δf" set to zero.

### Crystal data

**Table:**

C_6_H_8_NO^+^·C_7_H_3_N_2_O_6_^−^	*F*(000) = 332
*M**_r_* = 321.25	*D*_x_ = 1.532 Mg m^−^^3^
Monoclinic, *P*2_1_	Mo *K*α radiation, λ = 0.71073 Å
Hall symbol: P 2yb	Cell parameters from 7134 reflections
*a* = 9.4988 (19) Å	θ = 27.5–3.4°
*b* = 6.0803 (12) Å	µ = 0.13 mm^−^^1^
*c* = 12.109 (2) Å	*T* = 297 K
β = 95.21 (3)°	Block, colorless
*V* = 696.5 (2) Å^3^	0.35 × 0.22 × 0.20 mm
*Z* = 2	

### Data collection

**Table:**

Rigaku Mercury2 diffractometer	1733 independent reflections
Radiation source: fine-focus sealed tube	1209 reflections with *I* > 2σ(*I*)
Graphite monochromator	*R*_int_ = 0.098
Detector resolution: 8.366 pixels mm^-1^	θ_max_ = 27.5°, θ_min_ = 3.4°
ω and φ scans	*h* = −12→12
Absorption correction: multi-scan (CystalClear; Rigaku, 2005)	*k* = −7→7
*T*_min_ = 0.967, *T*_max_ = 0.975	*l* = −15→15
7134 measured reflections	

### Refinement

**Table:**

Refinement on *F*^2^	Secondary atom site location: difference Fourier map
Least-squares matrix: full	Hydrogen site location: inferred from neighbouring sites
*R*[*F*^2^ > 2σ(*F*^2^)] = 0.084	H-atom parameters constrained
*wR*(*F*^2^) = 0.218	*w* = 1/[\s^2^(*F*_o_^2^) + (0.0791*P*)^2^ + 1.2502*P*] where *P* = (*F*_o_^2^ + 2*F*_c_^2^)/3
*S* = 1.05	(Δ/σ)_max_ < 0.001
1733 reflections	Δρ_max_ = 0.38 e Å^−^^3^
210 parameters	Δρ_min_ = −0.28 e Å^−^^3^
1 restraint	Extinction correction: *SHELXL97* (Sheldrick, 2008), Fc^*^=kFc[1+0.001xFc^2^\l^3^/sin(2\q)]^-1/4^
Primary atom site location: structure-invariant direct methods	Extinction coefficient: 0.072 (14)

### Special details

**Table:**

Geometry. All esds (except the esd in the dihedral angle between two l.s. planes) are estimated using the full covariance matrix. The cell esds are taken into account individually in the estimation of esds in distances, angles and torsion angles; correlations between esds in cell parameters are only used when they are defined by crystal symmetry. An approximate (isotropic) treatment of cell esds is used for estimating esds involving l.s. planes.
Refinement. Refinement of *F*^2^ against ALL reflections. The weighted *R*-factor *wR* and goodness of fit *S* are based on *F*^2^, conventional *R*-factors *R* are based on *F*, with *F* set to zero for negative *F*^2^. The threshold expression of *F*^2^ > σ(*F*^2^) is used only for calculating *R*-factors(gt) *etc*. and is not relevant to the choice of reflections for refinement. *R*-factors based on *F*^2^ are statistically about twice as large as those based on *F*, and *R*- factors based on ALL data will be even larger.

### Fractional atomic coordinates and isotropic or equivalent isotropic displacement parameters (Å^2^)

**Table:**

	*x*	*y*	*z*	*U*_iso_*/*U*_eq_	
C1	0.3248 (7)	0.2505 (12)	−0.2268 (6)	0.0325 (16)	
C2	0.2992 (7)	0.1113 (12)	−0.1273 (6)	0.0314 (16)	
C3	0.2233 (7)	−0.0841 (11)	−0.1380 (5)	0.0294 (15)	
H1	0.1912	−0.1357	−0.2082	0.035*	
C4	0.1951 (8)	−0.2021 (12)	−0.0450 (6)	0.0323 (16)	
C5	0.2459 (8)	−0.1379 (13)	0.0596 (6)	0.0349 (16)	
H2	0.2290	−0.2195	0.1220	0.042*	
C6	0.3243 (7)	0.0559 (13)	0.0675 (5)	0.0320 (16)	
C7	0.3495 (7)	0.1844 (12)	−0.0223 (5)	0.0309 (15)	
H3	0.3988	0.3162	−0.0129	0.037*	
N1	0.1063 (7)	−0.3963 (12)	−0.0578 (6)	0.0427 (16)	
O1	0.3190 (6)	0.1545 (8)	−0.3201 (4)	0.0413 (14)	
O2	0.3450 (7)	0.4499 (9)	−0.2114 (5)	0.0498 (15)	
O3	0.0399 (7)	−0.4290 (12)	−0.1476 (5)	0.0618 (19)	
O4	0.1028 (7)	−0.5164 (11)	0.0228 (6)	0.0597 (18)	
N2	0.3724 (7)	0.1352 (13)	0.1794 (5)	0.0453 (18)	
O5	0.4282 (7)	0.3173 (11)	0.1871 (5)	0.0571 (17)	
O6	0.3546 (8)	0.0204 (12)	0.2592 (4)	0.063 (2)	
C8	0.2779 (8)	0.4903 (13)	0.4615 (6)	0.0352 (17)	
C9	0.1846 (9)	0.4540 (16)	0.3689 (6)	0.047 (2)	
H4	0.1890	0.3219	0.3306	0.056*	
C10	0.0863 (9)	0.6070 (18)	0.3322 (7)	0.054 (2)	
H5	0.0272	0.5818	0.2679	0.064*	
C11	0.0747 (9)	0.8013 (17)	0.3917 (7)	0.051 (2)	
H6	0.0045	0.9026	0.3691	0.062*	
C12	0.1687 (8)	0.8452 (15)	0.4857 (6)	0.0425 (18)	
H7	0.1630	0.9760	0.5248	0.051*	
C13	0.2689 (7)	0.6899 (13)	0.5182 (6)	0.0326 (15)	
O7	0.3797 (6)	0.3374 (10)	0.4957 (4)	0.0444 (14)	
H8	0.3601	0.2810	0.5539	0.067*	
N3	0.3724 (6)	0.7298 (10)	0.6120 (5)	0.0339 (14)	
H9	0.3537	0.8575	0.6435	0.051*	
H10	0.4585	0.7345	0.5887	0.051*	
H11	0.3683	0.6219	0.6613	0.051*	

### Atomic displacement parameters (Å^2^)

**Table:**

	*U*^11^	*U*^22^	*U*^33^	*U*^12^	*U*^13^	*U*^23^
C1	0.032 (4)	0.029 (4)	0.037 (4)	0.002 (3)	0.003 (3)	0.002 (3)
C2	0.033 (4)	0.030 (4)	0.032 (4)	0.001 (3)	0.007 (3)	−0.001 (3)
C3	0.032 (4)	0.029 (4)	0.028 (3)	−0.001 (3)	0.004 (3)	−0.003 (3)
C4	0.030 (4)	0.026 (4)	0.043 (4)	0.002 (3)	0.011 (3)	0.000 (3)
C5	0.037 (4)	0.034 (4)	0.034 (4)	0.005 (3)	0.009 (3)	0.007 (3)
C6	0.032 (4)	0.039 (4)	0.026 (3)	−0.003 (3)	0.003 (3)	−0.004 (3)
C7	0.026 (3)	0.031 (4)	0.035 (3)	0.001 (3)	0.003 (3)	−0.001 (3)
N1	0.033 (3)	0.038 (4)	0.059 (4)	−0.009 (3)	0.017 (3)	−0.004 (3)
O1	0.071 (4)	0.025 (3)	0.028 (3)	0.004 (3)	0.002 (2)	−0.002 (2)
O2	0.081 (4)	0.025 (3)	0.044 (3)	−0.007 (3)	0.010 (3)	0.000 (2)
O3	0.060 (4)	0.062 (4)	0.064 (4)	−0.032 (4)	0.004 (3)	−0.017 (3)
O4	0.059 (4)	0.041 (3)	0.082 (4)	−0.017 (3)	0.021 (3)	0.007 (4)
N2	0.039 (4)	0.064 (5)	0.032 (3)	−0.004 (4)	−0.001 (3)	−0.002 (3)
O5	0.069 (4)	0.050 (4)	0.052 (3)	−0.011 (4)	0.000 (3)	−0.010 (3)
O6	0.087 (5)	0.075 (5)	0.028 (3)	−0.019 (4)	0.003 (3)	0.001 (3)
C8	0.042 (4)	0.039 (4)	0.025 (3)	−0.005 (3)	0.008 (3)	−0.001 (3)
C9	0.058 (5)	0.049 (5)	0.033 (4)	−0.020 (5)	0.002 (4)	−0.007 (4)
C10	0.048 (5)	0.070 (7)	0.039 (4)	−0.010 (5)	−0.013 (4)	−0.001 (4)
C11	0.044 (5)	0.056 (6)	0.053 (5)	0.003 (4)	−0.004 (4)	0.010 (4)
C12	0.046 (5)	0.034 (4)	0.046 (4)	0.002 (4)	−0.001 (3)	−0.003 (4)
C13	0.030 (3)	0.032 (4)	0.036 (4)	−0.005 (3)	0.006 (3)	0.004 (3)
O7	0.059 (4)	0.039 (3)	0.035 (3)	0.008 (3)	0.005 (2)	0.001 (3)
N3	0.040 (3)	0.027 (3)	0.035 (3)	−0.001 (3)	0.006 (2)	0.000 (3)

### Geometric parameters (Å, º)

**Table:**

C1—O2	1.239 (9)	N2—O5	1.228 (10)
C1—O1	1.269 (8)	C8—O7	1.378 (9)
C1—C2	1.510 (10)	C8—C9	1.382 (10)
C2—C7	1.390 (9)	C8—C13	1.401 (10)
C2—C3	1.390 (10)	C9—C10	1.364 (14)
C3—C4	1.383 (9)	C9—H4	0.9300
C3—H1	0.9300	C10—C11	1.393 (14)
C4—C5	1.370 (10)	C10—H5	0.9300
C4—N1	1.451 (10)	C11—C12	1.407 (11)
C5—C6	1.393 (11)	C11—H6	0.9300
C5—H2	0.9300	C12—C13	1.373 (11)
C6—C7	1.377 (9)	C12—H7	0.9300
C6—N2	1.472 (9)	C13—N3	1.453 (9)
C7—H3	0.9300	O7—H8	0.8200
N1—O4	1.222 (9)	N3—H9	0.8900
N1—O3	1.223 (9)	N3—H10	0.8900
N2—O6	1.216 (9)	N3—H11	0.8900
			
O2—C1—O1	125.2 (7)	O5—N2—C6	117.5 (7)
O2—C1—C2	117.6 (7)	O7—C8—C9	121.1 (7)
O1—C1—C2	117.1 (6)	O7—C8—C13	120.6 (6)
C7—C2—C3	119.4 (6)	C9—C8—C13	118.2 (8)
C7—C2—C1	118.9 (6)	C10—C9—C8	121.6 (8)
C3—C2—C1	121.6 (6)	C10—C9—H4	119.2
C4—C3—C2	120.3 (6)	C8—C9—H4	119.2
C4—C3—H1	119.9	C9—C10—C11	119.6 (7)
C2—C3—H1	119.9	C9—C10—H5	120.2
C5—C4—C3	121.8 (7)	C11—C10—H5	120.2
C5—C4—N1	118.9 (6)	C10—C11—C12	120.3 (8)
C3—C4—N1	119.2 (6)	C10—C11—H6	119.8
C4—C5—C6	116.4 (6)	C12—C11—H6	119.8
C4—C5—H2	121.8	C13—C12—C11	118.3 (8)
C6—C5—H2	121.8	C13—C12—H7	120.8
C7—C6—C5	123.8 (6)	C11—C12—H7	120.8
C7—C6—N2	118.6 (7)	C12—C13—C8	121.8 (7)
C5—C6—N2	117.3 (6)	C12—C13—N3	120.8 (7)
C6—C7—C2	118.1 (7)	C8—C13—N3	117.5 (6)
C6—C7—H3	121.0	C8—O7—H8	109.5
C2—C7—H3	121.0	C13—N3—H9	109.5
O4—N1—O3	124.3 (7)	C13—N3—H10	109.5
O4—N1—C4	117.3 (7)	H9—N3—H10	109.5
O3—N1—C4	118.4 (7)	C13—N3—H11	109.5
O6—N2—O5	123.2 (7)	H9—N3—H11	109.5
O6—N2—C6	119.3 (7)	H10—N3—H11	109.5
			
O2—C1—C2—C7	−23.9 (10)	C5—C4—N1—O3	166.3 (7)
O1—C1—C2—C7	158.5 (6)	C3—C4—N1—O3	−12.2 (10)
O2—C1—C2—C3	154.2 (7)	C7—C6—N2—O6	−177.8 (7)
O1—C1—C2—C3	−23.4 (10)	C5—C6—N2—O6	7.0 (10)
C7—C2—C3—C4	1.5 (10)	C7—C6—N2—O5	2.8 (10)
C1—C2—C3—C4	−176.6 (6)	C5—C6—N2—O5	−172.4 (7)
C2—C3—C4—C5	−3.2 (11)	O7—C8—C9—C10	−178.0 (8)
C2—C3—C4—N1	175.2 (6)	C13—C8—C9—C10	0.3 (12)
C3—C4—C5—C6	1.7 (10)	C8—C9—C10—C11	−2.8 (13)
N1—C4—C5—C6	−176.7 (6)	C9—C10—C11—C12	3.3 (13)
C4—C5—C6—C7	1.4 (10)	C10—C11—C12—C13	−1.5 (12)
C4—C5—C6—N2	176.3 (6)	C11—C12—C13—C8	−1.0 (11)
C5—C6—C7—C2	−3.0 (10)	C11—C12—C13—N3	177.6 (7)
N2—C6—C7—C2	−177.8 (6)	O7—C8—C13—C12	179.9 (7)
C3—C2—C7—C6	1.4 (10)	C9—C8—C13—C12	1.6 (11)
C1—C2—C7—C6	179.6 (6)	O7—C8—C13—N3	1.3 (9)
C5—C4—N1—O4	−13.4 (10)	C9—C8—C13—N3	−177.0 (7)
C3—C4—N1—O4	168.1 (7)		

### Hydrogen-bond geometry (Å, º)

**Table:**

*D*—H···*A*	*D*—H	H···*A*	*D*···*A*	*D*—H···*A*
O7—H8···O1^i^	0.82	1.78	2.603 (7)	179
N3—H9···O1^ii^	0.89	1.90	2.771 (8)	168
N3—H10···O7^iii^	0.89	2.02	2.870 (8)	159
N3—H11···O2^i^	0.89	1.89	2.763 (8)	166
C12—H7···O1^ii^	0.93	2.53	3.240 (9)	134

Symmetry codes: (i) *x*, *y*, *z*+1; (ii) *x*, *y*+1, *z*+1; (iii) −*x*+1, *y*+1/2, −*z*+1.
